# Supplementary material for: Functional Characterization of TkSRPP Promoter in Response to Hormones and Wounding Stress in Transgenic Tobacco
Source: Plants (Basel). 2023 Jan 5;12(2):252. doi: 10.3390/plants12020252 (PMC9866153; doi:10.3390/plants12020252)
Supplement: Supplementary file 1 [file plants-12-00252-s001.zip › Supplementary file 1.pdf]

```
>utg19387|GWHAAAA00015227.   86556
Length=86556

 Score = 3635 bits (4030),  Expect = 0.0
 Identities = 2138/2213 (97%), Gaps = 25/2213 (1%)
 Strand=Plus/Minus

Query  1      TGCCCACTACTTTGGAGGGAtttttttCAACTGCTCACTTGCAGCCTTATCATAAAAGCT  60
              |
Sbjct  66869  TGCCCACTACTTTGGAGGGATTTTTTTCAACTACTCACTTGCAGCCTTATCATAAAAGCT  66810

Query  61     AAATTCAGACATGAAATGCTCAAACTCGGGTATTGTGGTGGCAGTGGCACATTTCCATAA  120
              |
Sbjct  66809  AAATTCAGACATGAAATGCTCAAACTCGGGTATTGTGGTGGCAGTGGCACATTTCCATAA  66750

Query  121    ATAATCCTTGTACACTGTCGGCTTCCAAACCTTCTATCTAATGAGTAATTATAATATTAT  180
              |
Sbjct  66749  ATAATCATTGTACACTGTCGGCTTCCAAACCTTCT---TCATG-----TTTTGATAT---  66701

Query  181    ACCTTATTGCTACATATTGCTGGAATAAGTACCTGGAACATTGGGGAAGCGGCTACATAT  240
              |
Sbjct  66700  ATGTGTCTGAGACAAAA--CTTGT----GTTC--GG--CATGGGGGAAGCGGCTACATAT  66651

Query  241    TGCTGGAATAAGTACCTGGAACATTGGAGCATAAAAAGCACAAAACATTATAAAACTGAG  300
              |
Sbjct  66650  TGCTGGAATAAGTCCCTGTAACATTGGAGCATAAAAAGCACAAAACATTATAAAACTGAG  66591

Query  301    TAGAAATATTGAAATATAAAACTACAAGACATCATATAACACAGTAGAGAAAGTTTTACC  360
              |
Sbjct  66590  TAGAAATATTGAAATATAAAACTACAAGACATCATATAACACAATAGAGAAAGTTTTACC  66531

Query  361    TTTTGCCTATCGGTGATGAATGTGAAGTTTAAGTTTCCACTAATGCTCTAAGGGGAACCC  420
              |
Sbjct  66530  TTTTGCCTATCGGTGATGAATGTGAAGTTTGAGTTTCCACTAATGCGCTAAGGGGAACCC  66471

Query  421    GAGGGTCTGATTCTATTTGGCTTAGGATTTCTTTAGAGAGGAAAGTAGCTGTACAAGCTC  480
              |
Sbjct  66470  GAGGGGCTGTTTCTATTTGGCTTAGGATTTCTTTAGAGAGGAAAGTACCTGTACAAGCTC  66411

Query  481    TAAGCTTTCTACTTCTCAAGCACGAGTGGTTGTCATGACAAACCAATCAGATTCTTCATT  540
              |
Sbjct  66410  TAAGCTTTCTACTTCTCAAGCACGAGTGGTTGTCATGACAAACCAATCAGATTCTTCATT  66351

Query  541    TGACCTTGAAGCATGTAGGGCCCACTTGCAACCTTCCTTTTTGGAGTTCACACATTTGTC  600
              |
Sbjct  66350  TGACCTTGAAGCATGTAGGGCCCACTTGCAACCTTCTTTTTTGGAGTTCACACATTTGTC  66291

Query  601    CTTGCTTTTAGTTGTCCCAACAACTCCACTTGAAAGCGAGCCAACCACTACACCTCTACA  660
              |
Sbjct  66290  CTTGCTTTTAGTTGTCCCAACAACTCCACTTGAAAGCGAGCCAACCACTACACCTCTACA  66231

Query  661    TTTTGCCCGAAGTCTCAATTTGTCAtttttttCAAAATACAAATTTCTCCTAGTTTGTAT  720
              |
Sbjct  66230  TTTTGCCCGAAGTCTCAATTTGTCATTTTTTTCAAAATACAAATTTCTCCTAGTTTGTAT  66171

Query  721    TGCATGAGCATCTATGAGATCTTTTAATTCTTTCTTTGACTTGAACTTTTGCTCGACATA  780
              |
Sbjct  66170  TGCATGAGCATCTATGAGATCTTTTAATTCTTTCTTTGACTTGAACTTTTGCCCGACATA  66111

Query  781    AAAGCTTGCTTTGTGAACATTTCCAAGATTGCATTTCTTCTCCTTACCCAAATTCTTGAG  840
```

|

Sbjct 66110 AAAGCTTGCTTTGTGAACATTTCCAAGATTGCATTTCTTCTCCTTACCCAAATTCTTGAG 66051

Query 841 AACATTTCTTCTTCTCATGTCATCATCAGACCCTTCATCTAAAGAATCCCATCTTTCATT 900

|

Sbjct 66050 AACATTTCTTCTTCTCATGTCATCATCAGACCCTTCATCTAAAGAATCTCATCTTTCATT 65991

Query 901 ATCTATTACATCCAAATCCTCATCAACTTCTTCATCAGTAGCCCCTCCATCAATATGAAC 960

|

Sbjct 65990 ATCTATTACATCCAAATCCTCATCAACTTCTTCATCAGTAGCCCCTCCATCAATATGAAC 65931

Query 961 TTCACTTTCATCTCTATCAAGGTTTAGTGTGAAATCAGCCATATCAACCTCAACTTCTGG 1020

|

Sbjct 65930 TTCACTTTCATCTCTATCAAGGTTTAGTGTGAAATCAGCCATATCAACCTCAACTTCTGG 65871

Query 1021 TACATTATTCTCCTCGTCTACAAGGTCGTCTTCCTTTAAATCAGCACCTTCATCACCTTC 1080

|

Sbjct 65870 TACATTATTCTCCTCGTCTACAAGGTCGTCTTCCTCTAAATCAGCACCTTCATCACCTTC 65811

Query 1081 TTTTTCAGTTGGCTGGTCTTCATCACTCTCATCTTCAAGTGGCTGGTCTTCATCACTCTC 1140

|

Sbjct 65810 TTTTTCAGTTGGCTGGTCTTCATCACTCTCATCTTCAAGTAGCTGGTCTTCATCACTCTC 65751

Query 1141 AAAGAGCATCATTGACATATATTCTCCTATAGCTGATGGCATTTCATCTAGAAGAAAAAA 1200

|

Sbjct 65750 AAAGAGCATCATTGACATATATTCTCCTATAGCTGATGGCATTTCATCTAGAAGAAAAAA 65691

Query 1201 CATAGCATTAATATCATCAAATAAACCTG---aaaaaaatgtgtaaaaaaCCGTGTGCCC 1257

|

Sbjct 65690 CATAGCATTAATATCATCAAAGAAACCTGGAAAAAAAAGTGTGTAAAAAACCGTGTGCCC 65631

Query 1258 AATATTATCTAAATATGTGGGACCCAAATCATCACCACAAGTATACCAAGTTTATGTTTT 1317

|

Sbjct 65630 AACATTATCTAAATATGTGGGACCCAAATCATCACCACAAGTATACCAAGTTTATGTTTT 65571

Query 1318 TTAAATTTTGTTAATTGAAGTTAGTTAAACATCTATTATTGTTTTGAGTTTAATATAGAG 1377

|

Sbjct 65570 TTAAATTTTGTTAATTGAAGTTAGTTAAACATCTATTATTGTTTTGAGTTTAATATAGAG 65511

Query 1378 TAAATGCTGCCTTCTTCTATAAAAATTCGTAAAGAAATTTCATACACCAACGATTTTACT 1437

|

Sbjct 65510 TAAATGCTGCCTTCTTCTAGAAAAATTCGTAAAGAAATTTCATACACCAACGATTTTACT 65451

Query 1438 TAAGTGTCGTTCTCTATGAATTGGAAGTGGTAACAAATTGTTTTAAAGCTTTTTCACGAC 1497

|

Sbjct 65450 TAAGTGTCGTTCTCTATGAATTGGAAGTGGTAACAAATTGTTTTAAAGCTTTTTCACGAC 65391

Query 1498 TAATTTTAGTGTGTAAAGATGTTGCTTTTTGCTTTTTACTGTTGTCATTAACGATTAGAT 1557

|

Sbjct 65390 TCATTTTAGTGTGTAAAGATGTTGCTTTTTGCTTTTTACTGTTGTCATTAACGATTAGAT 65331

Query 1558 GTAAGAATTCATAAATAAGGTTTGAGATGCATATCGTGAAACCAGTTGATAGAAACAGAT 1617

|

Sbjct 65330 GTAAGAATTCATAAATAAGGTTTGAGATGCATATCGTGAAACCAGTTGATAGAAACAGAT 65271

Query 1618 ACGCAATGATAGAGAGACGCAACCAAGGAATACAGATACAAGCGATACACAGATGACGAT 1677

|

Sbjct 65270 ACGCAATGATAGAGAGACGCAACCAAGTAATACAGATACAAGCGATACAAGGATGACGAT 65211

Query 1678 ACAAGGATAGAGAACTAACCATTAGCGCCGGACATGTCGCCTGTTTGCCCGCCAGAGAAG 1737

|

```
Sbjct  65210  ACAAGGATAGAGAACTAACCATTCGCGCCGGACATGTCGCCTGTTTGCCCGCCAGAGAAG  65151

Query  1738   TAACGGGAGGGTTTCTGTGTTAAACATAGATCCCTGGAGGCGGGAAGACAAATTTGCCCT  1797
        |
Sbjct  65150  TAACGGGAGGGTTTCTGTGTTAAACATAGATCCCTGGAGGCGGAAAGACAAATTTGCCCT  65091

Query  1798   CGTGTGCAGTGCACATGAGGGTTTAACGGGTTTAGGTTAACGGGAGTTAAAGGTAGGGAC  1857
        |
Sbjct  65090  CGTGTGCAGTGCACATGAGGGTTTAACGGGTTTAGGTTAACGGGAGTTAAAGGCAGGGAC  65031

Query  1858   AGAAACCACAATGAAACATTGAAAGAATGACTGTTTATCCAAGTTTTAAAGTTAAGGGAC  1917
        |
Sbjct  65030  AGAAACCACAATGAAACATTTAAATAATGACTGTTTATCCAAGTTTTAAAGTTAAGGGAC  64971

Query  1918   TCAAACCATAG-TTTTTCCAAACCACATGGACtttttttttGCAGTTTTGTCAATATTTA  1976
        |
Sbjct  64970  TCAAACCACAGTTTTTTCCAAACCACAGGGACCTTTTTTTTGGAGTTTTGTCAATATTTT  64911

Query  1977   ATCAATCTATATTTTCATTATAATTATCGACACTCCTGGACCCACAGTAGTACAGTTAAA  2036
        |
Sbjct  64910  ATCAATCTATATTTTCATTATAATTATCGACACTCCTGGACCCACAGTAGTACGGTTAAA  64851

Query  2037   CATTTAGTTTTCGTGGAATTTATGCCCCTAAAACCCACTCTATATAAATAACGATGCATG  2096
        |
Sbjct  64850  CATTTAGTTTTCGTGGAATTTATGCCCCTAAAACCCACTCTATATAAATAACGATGCATG  64791

Query  2097   GACAGAGATATGAATACACTTTCTGATTTAAGCAACAATCATTTCTCGATAGACGACTTT  2156
        |
Sbjct  64790  GACAGAGATATGAATACACTTTCTGATTTAAGCAACAATCATTTCTCGATAGACGACTTT  64731
                                                 ↓
Query  2157  TCATCTCGTTTTGAACCATATACATCTTGATCATGACCGACGCTGCTTCTGTT  2209
        |
Sbjct  64730  TCATCTCGTTTTGAACCATATACATCTTGATCATGACCGACGCTGCTTCTGTT  64678
```
