# Supplementary material for: Functional Characterization of TkSRPP Promoter in Response to Hormones and Wounding Stress in Transgenic Tobacco
Source: Plants (Basel). 2023 Jan 5;12(2):252. doi: 10.3390/plants12020252 (PMC9866153; doi:10.3390/plants12020252)
Supplement: Supplementary file 1 [file plants-12-00252-s001.zip › Supplementary file 2.pdf]

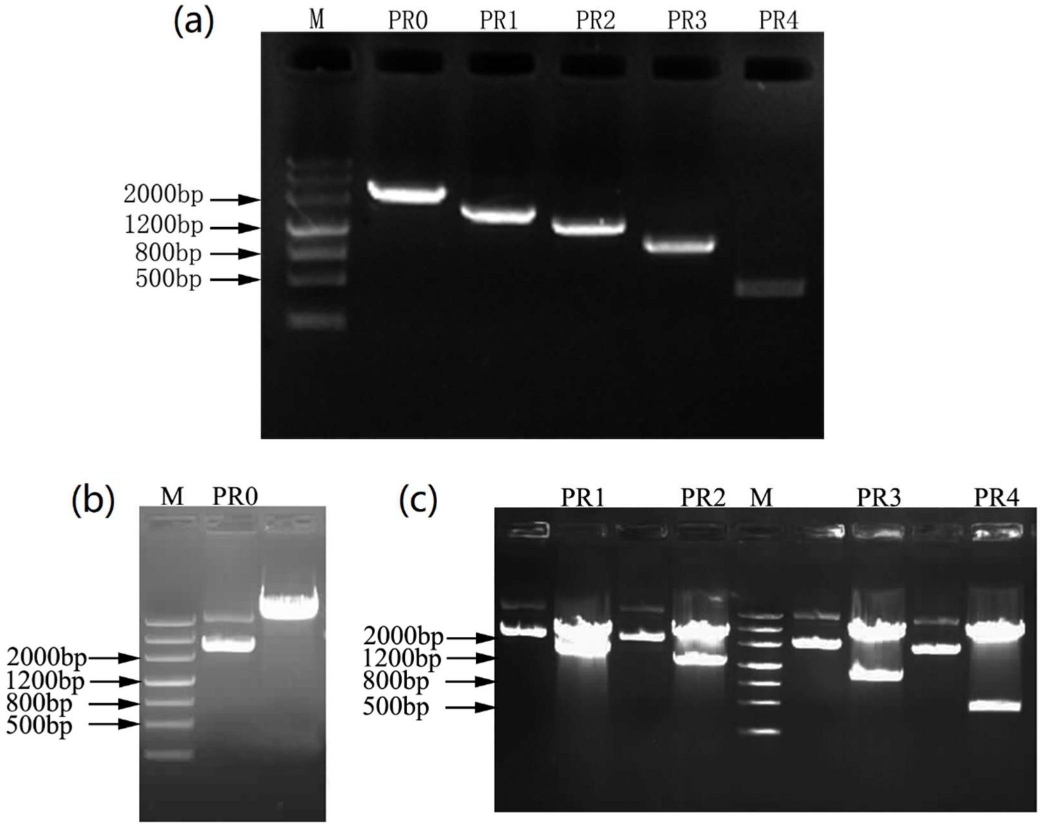

Figure S1

Cloning and double digests analysis of different *TkSRPP* promoters. (a): Cloning of the full-length *TkSRPP* promoter (PR0). (b): Double digests of the pCAMBIA1304-PR0::GUS recombinant plasmid. (c): Double digests of the pCAMBIA1304-PR1/PR2/PR3/PR4::GUS recombinant plasmid.

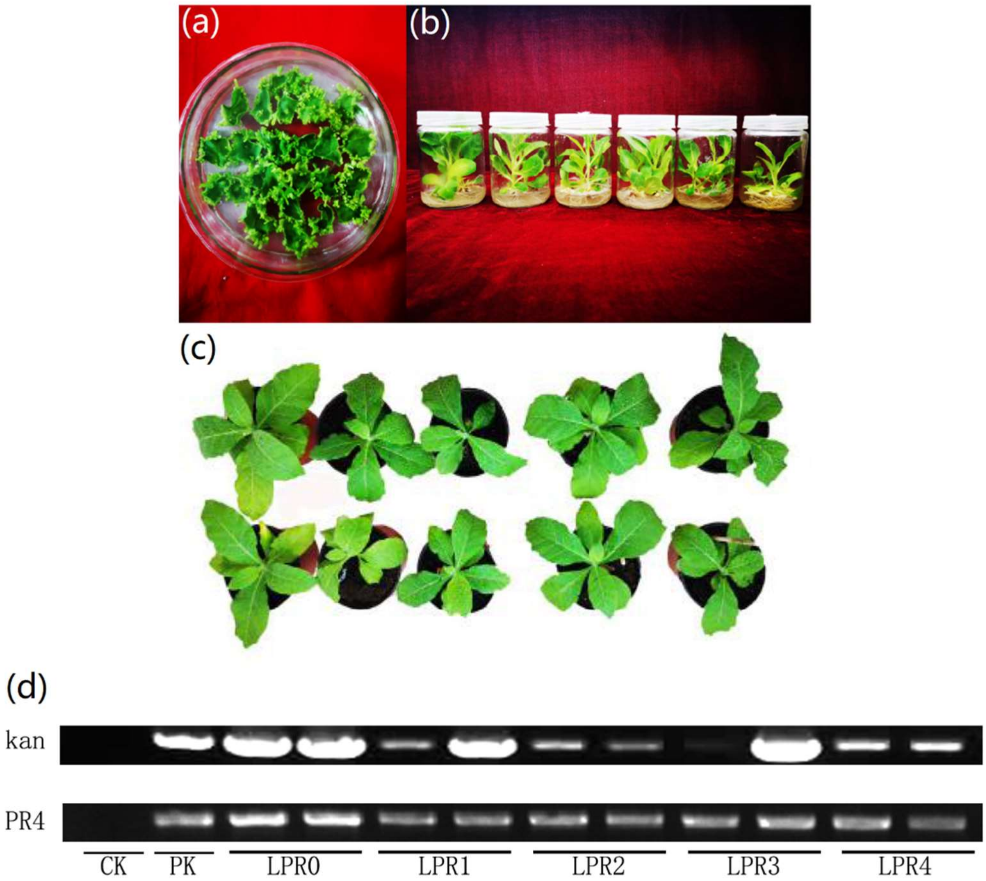

Figure S2

Genetic transformation and identification of transgenic tobacco with different deletion promoters (a): The callus of tobacco leaves differentiated into regenerative buds. (b): Rooting stage of tobacco regeneration buds. (c): The soil culture stage of transgenic tobacco. (d): Transgenic tobacco was identified using *Kan* gene primers and PR4 promoter primers, respectively.
