# Supplementary material for: Functional Characterization of TkSRPP Promoter in Response to Hormones and Wounding Stress in Transgenic Tobacco
Source: Plants (Basel). 2023 Jan 5;12(2):252. doi: 10.3390/plants12020252 (PMC9866153; doi:10.3390/plants12020252)
Supplement: Supplementary file 1 [file plants-12-00252-s001.zip › Supplementary file 3.pdf]

Table S1 Primer sequences of different *TkSRPP* promoter cloning

Primer name	Primer sequence (5'-3')
SRPP-FP	TGCCCACTACTTTGGAGGGATT
SRPP-RP	AACAGAAGCAGCGTCGGTCA
SRPP-FP0	GGGATCCCTGCCCACTACTTTGGAGGGATT
SRPP-FP1	GGGATCCCAACTCCACTTGAAAGCGAGCC
SRPP-FP2	GGGATCCCAGCCCCTCCATCAATATGAACTT
SRPP-FP3	GGGATCCCGGACCCAAATCATCACCACAAG
SRPP-FP4	GGGATCCCACATAGATCCCTGGAGGCGG
SRPP-RP0	CCCATGGGAACAGAAGCAGCGTCGGTCA

The underlined part form restriction sites: *Bam*H I（*GGGATCCC*）and *Noc* I（*CCCATGGG*）

Table S2 Identification of different SRPP promoter transgenic tobacco

Primer name	Primer sequence (5'-3')
NPT II-FP1	CACTGAAGCGGGAAGGGACT
NPT II-RP1	CGATACCGTAAAGCACGAGGAA
SRPP-FP4	ACATAGATCCCTGGAGGCGG
SRPP-RP0	AACAGAAGCAGCGTCGGTCA

Table S3 Quantitative Primer sequences of *TkSRPP* gene

Primer name	Primer sequence (5'-3')
SRPP-FP5	CCAGAAGCCTGTCAACTAACG
SRPP-RP5	TGGAACCAATGGCAGATAAGA
GAPDH-FP1	CGCCTCATTTAACATCATCC
GAPDH-RP1	GTCATACCACGAAACCAGCT

Table S4 Quantitative Primer sequences of *GUS* gene

Primer name	Primer sequence (5'-3')
GUS-FP1	CCAGAAGCCTGTCAACTAACG
GUS-RP1	TGGAACCAATGGCAGATAAGA
NbEF1-FP1	GATTGGTGGTAT TGGAACTGTCC
NbEF1-RP1	GAGCTTCGTGGTGCATCTC
